# Immunological response and implications of Ad26.COV2. S (Janssen COVID-19 vaccine) vaccine in diabetic patients: a prospective cohort study in Ethiopia

**DOI:** 10.3389/fimmu.2025.1546114

**Published:** 2025-05-08

**Authors:** Chala Kenenisa Edae, Abdisa Tufa Bedada, Maria Degef Teklemariam, Abera Botore Gudisa, Abdurahman Adamu, Solomon Genet Gebre

**Affiliations:** ^1^ Department of Biochemistry, School of Medicine, College of Health Sciences, Addis Ababa University, Addis Ababa, Ethiopia; ^2^ Public Health Emergency and Management Directorate, Oromia Health Bureau, Addis Ababa, Ethiopia; ^3^ Center for Advanced Research, Education and Training (CAMRET), Usmanu Danfodiyo University of Sokoto (UDUS), Sokoto, Nigeria

**Keywords:** diabetes mellitus, COVID-19, Ad26.COV2.S, Janssen vaccine, immune response, Ethiopia, immunoglobulins, inflammation

## Abstract

**Introduction:**

Patients with Diabetes are at increased risk of severe COVID-19 and death, thus, it is imperative to provide them with vaccination. Ad26.COV2. S vaccine has proven its efficacy. However, the immunological response of the patients with diabetes in Ethiopia has not been well studied.

**Methods:**

This prospective cohort study assessed immune responses after vaccination with a single dose of the Ad26.COV2.S. The subjects were enrolled diabetic patients who were 18 years old and above and attended a diabetes clinic at Adama Hospital Medical College. A sufficient blood sample was collected from each participant, following established standard protocols. We evaluated correlations among selected immunological parameters (IgG, IgM, CRP, IL6, IFN-Y) and employed statistical techniques such as chi-square tests, independent t-tests, and Generalized Estimating Equations (GEE) to analyze differences between given vaccinated and non-vaccinated cohorts. Generalized Estimating Equations (GEE) are a statistical method for modeling longitudinal or clustered data, particularly useful when dealing with non-normal data like binary or count data, by estimating parameters of a generalized linear model while accounting for potential correlations between observations.

**Results:**

It was found that vaccinated subjects showed significant alterations in the immune response with IgM elevation and a temporary increase of inflammatory biomarkers CRP and IL-6. Younger age and females were associated with lower inflammatory markers, and no significant effects of lifestyle factors (alcohol, chat, smoking) on immunological outcomes were observed. This vaccine elicited significant immunological responses in diabetic patients, characterized by initial increases in inflammatory markers and subsequent stabilization, and with implications for the healthcare policies to design tailored approaches for diabetic groups.

## Introduction

Diabetes mellitus is one of the leading global health problems, and millions across the globe suffer from it, with approximately 463 million people living with diabetes to date. This gives rise to serious comorbidities, especially in combination with infectious diseases like COVID-19 (1). On average, compared to those without diabetes, patients with diabetes have a higher risk of severe disease and increased hospital stay, as well as case-fatality rates at 7.3% for hospitalized COVID-19 subjects having been also infected by SARS-CoV-2. Consequently, rapid progress is critical, highlighting that safe vaccination strategies should be implemented in these vulnerable groups 2). Worldwide, more than 11 billion COVID-19 vaccine doses have been given to control the pandemic. Ad26.COV2.S is a top contender with its one-shot approach that helps address vaccine hesitancy, increasing coverage and showing efficacy in clinical trials (3). However, the immunological response to this vaccine in diabetic patients, particularly regarding the durability of antibody responses and potential side effects in diverse populations such as those in Ethiopia, remains insufficient (4).

Like many other low- and middle-income countries (LMICs), Ethiopia is dealing with unique challenges while managing diabetes along with COVID-19 concurrently. Large limitations in healthcare resources, namely a doctor-to-patient ratio of 1:10,000 and variable vaccine availability, have worsened the problem (5). Although COVID-19 vaccination is prioritized in diabetic patients, crucial evidence gaps exist concerning immunologic changes and, overall, the Ad26.COV2.S response across this population concerning a ‘real-world scenario’ context of Ethiopia (6). Hence, this prospective cohort study was designed to assess the immunological changes after COVID-19 vaccination with Ad26.COV2.S at Adama Hospital Medical College among diabetic patients in the Oromia region of Ethiopia. The effectiveness of the vaccine was evaluated for induced immunity to provide important real-world evidence reflecting immune responses in clinical practice. This will also provide an evidence-based perspective for future healthcare strategies and clinical practices, which may even guide policies on the frequency of booster doses and overall management of COVID-19 in diabetes mellitus patients that can further assist public health efforts better.

## Methods

### Study design and setting

A prospective cohort study to assess immune responses post-vaccination with the Ad26.COV2.S in patients with diabetes. The research was carried out at Adama Hospital Medical College, Oromia, Ethiopia, for 1 year (May 01/2023–June 30/2024). Baseline, three months after vaccination, and six, nine-, and twelve-months post-vaccination, immunological markers were determined. It was done among diabetes mellitus patients from Adama Hospital Medical College as the study population. On subsequent visits, follow-ups were made for blood sample collection and analysis of major immunological markers including IgG, IgM, C-reactive protein (CRP), interleukin-6(IL-6) & interferon-gamma (IFN-γ). Diabetic group (Unvaccinated Patients): A cohort of unvaccinated diabetic patients served as the control for comparative analysis.

### Study population

To achieve this objective, we conducted research among Type 2 diabetes patients aged ≥18 years attending the diabetic clinic at Adama Hospital Medical College. Patients were included based on a diagnosis of Type 2 diabetes mellitus according to established guidelines and subsequently attended the hospital for follow-up. Patients aged≥18 years old who did not have a history of severe allergic reactions to vaccines, immunodeficiency or autoimmune disease requiring systemic therapy, or pregnancy with an indication for vaccination in the third trimester were included. Socio-demographic and clinical data levels and blood samples were collected at baseline time before vaccination was done and subsequently after post-vaccination follow-up one year in three-month intervals.

### Sample size

The sample size for the study is determined by considering the longitudinal (repeated measures from each subject over time) nature of the research. The sample size is calculated using the formula from Diggle et al. (7). Five measurements were planned in the study, which occurred at baseline, three months, six months, nine months, and one year. The following parameters were used for sample size determination: The number of time points (t = 5), type I error rate (α = 0.05), power (90%), smallest meaningful difference (d = 0.5), and a ratio of 1: 3 (λ = 3) between the vaccinated and unvaccinated groups. We assumed a standard deviation (σ) of 1 and a correlation (r = 0.02) of repeated measures. The unequal sample size between the groups is due to various reasons. The expected effect size is small (the 0.5 mean difference observed in our study). Having a larger control group (unvaccinated individuals) can enhance the precision of our estimates and increase statistical power. During the initial COVID-19 vaccination season, unvaccinated individuals were more readily available due to low vaccination coverage. Additionally, we anticipated that many of these unvaccinated individuals may choose to get vaccinated and drop out of the study during the follow-up period.

A minimum sample size of 13 vaccinated and 39 unvaccinated participants was required. To account for potential attrition, a 10% adjustment was added to the minimum required sample sizes; therefore, 15 vaccinated and 45 unvaccinated participants were needed. The formula used for the calculation of sample size is expressed below:


$$\text{nt}=({(\text{λ}+1)\;}^{*}{\;(\text{Z}\_(1-\text{α}/2)+\text{Z}\_\text{β})}^{\wedge }2^{*}{\;({\text{σ}}^{\wedge }2)}^{*}(1+{\left(\text{t}-1\right)}^{*}\text{r}))/({\text{λ}}^{*}{\text{d}}^{*}{\left(\text{μ}1-\text{μ}2\right)}^{\wedge }2)$$

Particularly, given our continuous counseling efforts and ongoing national vaccination campaigns, we initially recruited 75 vaccinated and 225 unvaccinated participants who met the inclusion criteria. However, during the follow-up period, 18 participants in the vaccinated group and 58 in the unvaccinated group were lost due to vaccination uptake or other factors. Ultimately, 57 vaccinated and 167 unvaccinated participants completed the study, which remained above the minimum required sample size for the study.

### Data collection

Structured interviews and a review of hospital records were undertaken to collect baseline data including demographic information, clinical history [irrespective of whether patients presented at the first episode or recurrence (relapse)], lifestyle factors, e.g. smoking status, alcohol intake consumption, and khat use among others. Blood samples to analyze parameters for immunological responses were collected at baseline, three, six, nine, and twelve months post-vaccination.

### Laboratory analysis

A standard blood draw was obtained at every follow-up, according to established protocols. The levels of IgG, IgM, C-reactive protein (CRP), interleukin-6(IL-6), and interferon-gamma (IFN-γ) in serum were quantitatively detected by the ELISA method. These biomarkers were selected for their relevance in evaluating the immune response following vaccination. Blood samples were stored appropriately and analyzed at the Adama Public Health Research and Referral Laboratory Center in Adama, Ethiopia, and subsequently analyzed at the Center for Advanced Medical Research, Education, and Training (CAMRET) at Usmanu Danfodiyo University in Sokoto, Nigeria, according to a signed and approved material transfer agreement. Transport was conducted strictly to sample storage, packaging, and transfer guidelines.

### Statistical analysis

Analysis of data was performed by utilizing SPSS 26.0 and STATA 18 software, taking advantage of their advanced statistical features. Descriptive statistics were used to summarize the basic facts, such as age, gender, and the drugs given. The Chi-square tests were used to assess variations in categorical variables, whereas independent t-tests were employed to analyze continuous variables, like antibody levels, among varying groups. Generalized Estimating Equations (GEE) were used to assess longitudinal changes in outcome measures, accounting for within-subject correlations over time. Effect sizes, specifically Cohen’s d, were calculated to assess clinical significance. Statistical significance was set at p < 0.05, which shows significant differences among various groups. Generalized Estimating Equations (GEE) are a statistical method for modeling longitudinal or clustered data, particularly useful when dealing with non-normal data like binary or count data, by estimating parameters of a generalized linear model while accounting for potential correlations between observations.

### Ethical considerations

This study was approved by the Institutional Review Board of the Addis Ababa University College of Health Sciences (approval protocol number: 019/23/biochemistry) and the National Research Ethics Review Committee of the Ministry of Education (Ref No: 17/152/235/24). Written informed consent was received from all participants after explaining the purpose, risks, and benefits of the study during data and sample collection. This study strictly adhered to the principles of the Declaration of Helsinki, including respect for autonomy, beneficence, and justice.

## Results

### Comparative response of the immune system to the vaccine

A chi-square test of association was done to compare the baseline characteristics of study participants according to their vaccination status for several demographic and health-related factors, including age, sex, alcohol use, chat use, smoking, and medication types. Accordingly, there were no statistically significant differences between vaccinated and unvaccinated participants across these characteristics (p > 0.05). As shown in **Table 1**, among the participants, 143 (63.8%) were over 40 years old, with a mean age of 46.2 years (standard deviation: 11.6 years), ranging from 24 to 70 years. Specifically, 23 (28.1%) of those aged ≤40 years and 34 (23.8%) of those aged >40 years were vaccinated.

**Table 1:** Baseline characteristics of study participants by vaccination status (N=224).

Variables	Categories	Vaccination Status (n, %)	P-value	χ²	Row Percentages Vaccinated (n=57)	Row Percentages Unvaccinated (n=167)
**Age**	<=40	23 (28.4)	.446	0.580	28.4%	71.6%
	>40	34 (23.8)			23.8%	76.2%
	**Total**	57 (25.5)				
**Sex**	Female	29 (25.2)	.936	0.019	25.2%	74.8%
	Male	28 (25.7)			25.7%	74.3%
**Medications**	Insulin	13 (23.2)	.399	0.070	23.2%	76.8%
	Metformin	24 (22.9)			22.9%	77.1%
	Both	20 (31.8)			31.8%	68.3%
**Mean Age**		43.2 ± 12.6				

Bold text indicates statistically significant results (p < 0.05).

As shown in **Table 2**, an independent t-test was used to analyze the differences between vaccinated and unvaccinated groups regarding the outcome variables while considering the effect of time. Effect sizes were computed for the vaccinated group to assess the clinical significance of the observed differences. Statistical significance was determined at p < 0.05. At baseline, only C-reactive protein (CRP) and interleukin 6 (IL6) showed statistically significant differences between the two groups, with the unvaccinated group exhibiting slightly higher values. In general, the vaccinated group demonstrated lower values across most outcome variables compared to the unvaccinated group.

**Table 2:** Comparison of outcomes between vaccinated and unvaccinated groups over time (Mean [95% CI] and Effect Size).

Outcomes	Baseline	Effect Size	3 Months	Effect Size	6 Months	Effect Size	9 Months	Effect Size	1 Year	Effect Size
	Vaccinated vs Unvaccinated		Vaccinated vs Unvaccinated		Vaccinated vs Unvaccinated		Vaccinated vs Unvaccinated		Vaccinated vs Unvaccinated	
**IgG**	2.35 (-25.25, 29.96), p = .866	0.02	8.99 (-13.89, 31.88), p = .439	0.08	9.96 (-9.79, 29.72), p = .323	0.09	3.09 (-16.20, 22.39), p = .752	0.03	7.46 (-11.75, 26.67), p = .445	0.07
**IgM**	0.38 (-2.13, 2.88), p = .767	0.04	3.50 (1.11, 5.89), p <.001*	0.43	2.14 (-.21, 4.49), p = .075	0.27	-0.42 (-2.84, 1.99), p = .733	-0.05	-1.10 (-3.51, 1.30), p = .368	-0.14
**CRP**	-0.36 (-0.58, -0.14), p <.001*	-0.50	3.25 (3.13, 3.36), p <.001*	4.49	1.95 (1.83, 2.06), p <.001*	2.69	0.95 (0.83, 1.06), p <.001*	1.31	-1.25 (-1.37, -1.14), p <.001*	-1.74
**IFN**	-0.27 (-2.59, 2.06), p = .820	-0.04	16.83 (14.02, 19.64), p <.001*	1.88	2.13 (-0.31, 4.57), p = .086	0.27	-3.36 (-5.68, -1.04), p = .005*	-0.43	-3.36 (-5.68, -1.04), p = .005*	-0.43
**IL6**	-0.47 (-0.75, -0.18), p = .002*	-0.47	8.83 (8.37, 9.29), p <.001*	8.98	7.69 (7.24, 8.15), p <.001*	7.83	5.49 (5.04, 5.95), p <			

An asterisk (*) denotes statistically significant results (p <.05).

Bold text indicates statistically significant results (p < 0.05).

### Correlation analysis among immunological and metabolic variables

As shown in **Table 3**, independent correlation coefficients among various outcome variables highlight the relationships between immunological markers (IgG, IgM, CRP, IFN, IL6). Significant correlations (marked with an asterisk for p< 0.05) are provided in the table. Major findings include a strong positive correlation between CRP and IL6 (r = 0.79), showing a robust association between these inflammatory markers. IgM also exhibited significant positive correlations with several other variables, including CRP (r = 0.29) and IL-6 (r = 0.20), suggesting a link between IgM levels and inflammation disturbances.

**Table 3:** Independent correlation coefficients among outcome variables.

Variables	IgG	IgM	CRP	IFN	IL6
**IgG**	1.00				
**IgM**	.18*	1.00			
**CRP**	.13*	.29*	1.00		
**IFN**	-.01	-.01	.23*	1.00	
**IL6**	.12*	.20*	.79*	.19*	1.00

*p-value<0.05.

Bold text indicates statistically significant results (p < 0.05).

### Immunoglobulin G average value trends

**Figure 1:**
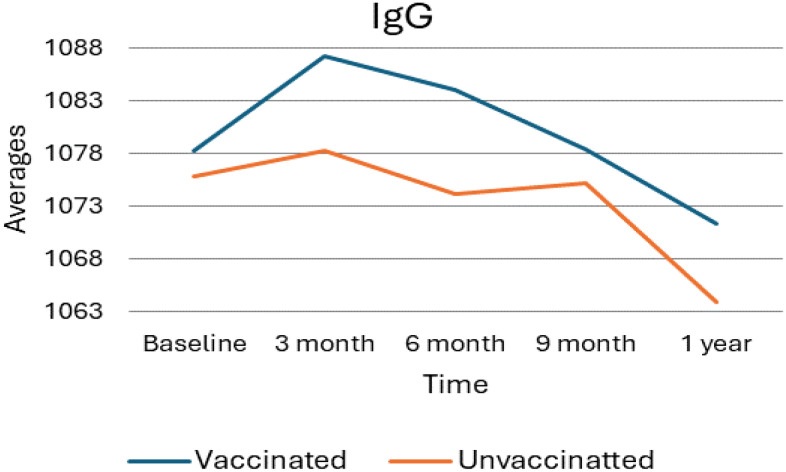
Average IgG levels among vaccinated and unvaccinated groups over time. Image shows the average IgG levels for both vaccinated and unvaccinated groups across various time points. The x-axis represents the follow-up time (baseline, 3 months, 6 months, and 9 months), whereas the y-axis displays the average IgG concentration. In general, the graph highlights the trend of IgG changes, showing an increase at 3 months followed by a decline, with the unvaccinated group experiencing a modest rise at 9 months.

As shown in the following **Figure 1**, the average levels of Immunoglobulin G (IgG) were analyzed for both vaccinated and unvaccinated groups at each follow-up time. During the third month, IgG levels increased in both groups compared to baseline; however, levels decreased during subsequent follow-up periods. Considerably, in the unvaccinated group, there was a minor increment in IgG levels at nine months compared to six months. In general, the vaccinated group consistently exhibited higher IgG levels than the unvaccinated group all over the study period.

### Generalized estimating equation results for IgG analysis

As **Table 4** indicates, a total of eight variables were considered for the analysis, with a simple GEE regression performed to select potential candidates for the GEE multiple regression. Variables with a p-value <=.20 were considered for inclusion in the multiple regression. For the analysis of IgG levels, six variables, including the time-treatment interaction, were examined. Three of these variables were found to be statistically significant (p-value < 0.05); The vaccination status did not exhibit a statistically significant effect on the IgG profile and Time, age group, and alcohol exposure. A significant difference was noted between baseline and one-year measurements, with a corresponding negative coefficient of -10.70, indicating that IgG levels significantly decreased at one year compared to baseline. For the age group (<=40 years), it was negative, suggesting a decrement of -19.02 in IgG levels relative to older counterparts. The coefficient associated with alcohol exposure (yes) was also negative, indicating a decrement of -27.55 in IgG profiles for those exposed to alcohol.

**Table 4:** GEE multiple regression for IgG crude and adjusted effects.

Variables (N=224)	Crude Effect	Adjusted Effect
	β (95% CI)	P-Value
Vaccination Status		
Vaccinated	6.37 (-7.64, 20.39)	.373 ^bc^
Unvaccinated	Ref.	
**Time**		<.001**
3 months	4.03 (-.89, 8.96)	.109
6 months	.15 (-19.87, 20.18)	.988
9 months	-.45 (-20.43, 19.54)	.965
1 year	-10.70 (-16.67, -4.73)	<.001**
Baseline	Ref.	
Group*Time		
NA	NA	.916
Vaccinated * 3 months		6.64 (-12.45, 25.74)
Vaccinated * 6 months		7.61 (-27.06, 42.28)
Vaccinated * 9 months		.74 (-33.95, 35.43)
Vaccinated * 1 year		5.11 (-18.33, 28.54)
Vaccinated * Baseline	Ref.	
Age Group		
<=40 years	-19.22 (-34.15, -4.29)	.012**
>40 years	Ref.	
Sex		
Female	13.73 (-1.86, 29.32)	.084**
Male	Ref.	
Alcohol Exposure		
Yes	-27.74 (-46.96, -8.53)	.005**
No	Ref.	
Chat Exposure		
Yes	2.31 (-18.12, 22.74)	.825
No	Ref.	
Smoking Exposure		
Yes	5.71 (-60.50, 71.92)	.866
No	Ref.	
**Medications**		.869
Insulin	-5.69 (-26.76, 15.37)	.596
Metformin	-2.74 (-21.64, 16.16)	.776
Both	Ref.	
**Intercept**	1088.98	

Bold text indicates statistically significant results (p < 0.05).

**P- value ≤ .20; * p-value < 0.05.

bc, biological criteria; NA, Not Applicable.

### Immunoglobulin M average value trend

**Figure 2:**
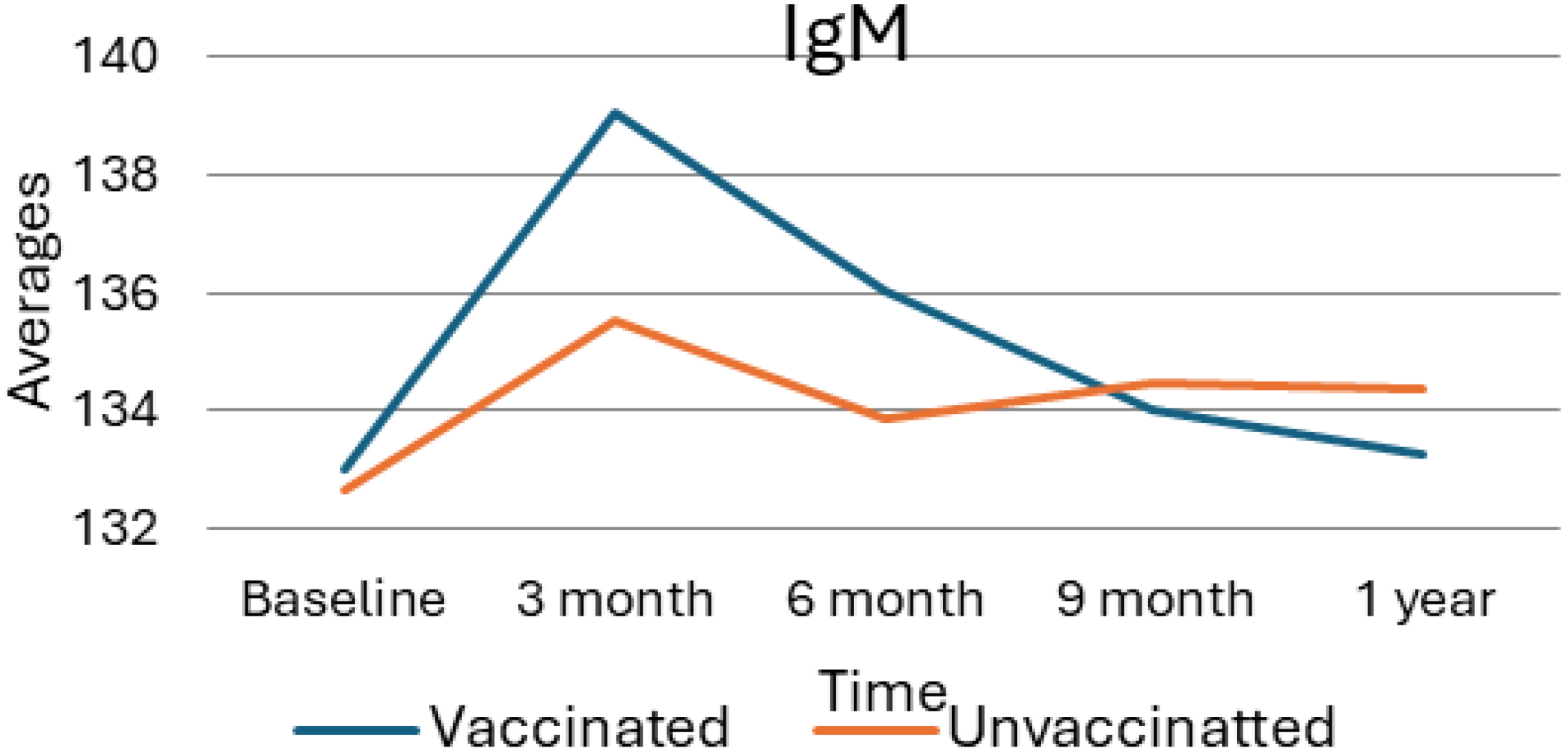
Average value of IgM for vaccinated and unvaccinated groups during various time points. The above Image shows the average IgM levels for both vaccinated and unvaccinated groups across various time points. The x-axis represents the follow-up time (baseline, 3 months, 6 months, and 9 months), whereas the y-axis displays the average IgM concentration. In general, the graph highlights the trend of IgM changes, showing an increase at 3 months followed by a decline.

As presented in the following **Figure 2**, the average levels of Immunoglobulin M (IgM) in vaccinated versus unvaccinated individuals over one year, with measurements taken at baseline, 3 months, 6 months, 9 months, and 1-year post-vaccination. At the initial or baseline, both groups demonstrate similar IgM levels and, at the three-month mark, a notable increase is observed in the vaccinated group, reaching an average IgM level of approximately 139. In contrast, the unvaccinated group shows a modest rise to about 135. As the study progresses, IgM levels in both groups decline; however, the vaccinated group exhibits a sharper decrease. By the nine-month mark, the IgM levels of both groups aligned closely, and this trend continued, with slight declines noted at the one-year follow-up. Overall, these findings indicate a temporary enhancement of the immune response in the vaccinated group, which tapers off over time, suggesting that the initial boost in IgM levels following vaccination eventually normalizes and approximates the levels observed in the unvaccinated group.

### GEE multiple regression of IgM – crude and adjusted effects

**Table 5** summarizes the results of the Generalized Estimating Equations (GEE) multiple regression analysis, which assessed both the crude and adjusted effects of several factors on IgM levels among a sample of 224 participants. The analysis investigated the influence of vaccination status, time points, the interaction between group and time, age, sex, and various exposures (alcohol, chat, smoking) and medication use on IgM levels. The result remained consistent in the adjusted analysis (β = 0.68, 95% CI: -1.12, 2.49; p = 0.457), which suggests no significant difference in IgM levels between vaccinated and unvaccinated individuals when controlling for other variables. Time was identified as a significant predictor of IgM levels in both crude and adjusted models. These results indicate significant fluctuations in IgM levels over time, particularly in the months following vaccination. Overall, no significant effects were observed for sex, alcohol, chat, or smoking exposure. Medication did not demonstrate a statistically significant effect on IgM levels in the adjusted model. The results of the GEE multiple regression analysis indicate that time and age are key determinants of IgM levels in this sample. Significant changes in IgM levels were particularly evident in the early months following vaccination, while vaccination status alone did not produce significant differences in overall IgM levels. These findings suggest that temporal dynamics and age-related factors should be considered when evaluating immune responses in vaccinated populations.

**Table 5:** GEE multiple regression of IgM crude and adjusted effects.

Variables (N=224)	Crude Effect	Adjusted Effect
	β (95% CI)	P-Value
Vaccination Status		
Vaccinated	.89 (-1.18, 2.98)	.397 bc
Unvaccinated	Ref.	Ref.
**Time**		<.001**
3 months	3.67 (2.56, 4.78)	<.001**
6 months	1.69 (.42, 2.95)	.009
9 months	1.59 (.34, 2.85)	.013
1 year	1.35 (.08, 2.62)	.037
Baseline	Ref.	Ref.
Group*Time	NA	<.001*
Vaccinated * 3 months	3.13 (1.65, 4.59)	<.001*
Vaccinated * 6 months	1.76 (.07, 3.46)	.042*
Vaccinated * 9 months	-.79 (-2.48,.88)	.353
Vaccinated * 1 year	-1.48 (-3.31,.35)	.113
Vaccinated * Baseline	Ref.	Ref.
Age Group		
<=40 years	-5.43 (-6.92, -3.94)	<.001**
>40 years	Ref.	Ref.
Sex		
Female	.61 (-1.03, 2.26)	.464
Male	Ref.	Ref.
Alcohol Exposure		
Yes	-1.05 (-2.94,.83)	.274
No	Ref.	Ref.
Chat Exposure		
Yes	-.39 (-2.86, 2.09)	.758
No	Ref.	Ref.
Smoking Exposure		
Yes	2.52 (-4.61, 9.65)	.488
No	Ref.	Ref.
**Medications**		.179**
Insulin	.51 (-1.88, 2.89)	.677
Metformin	1.76 (-.31, 3.84)	.095
Both	Ref.	Ref.
**Intercept**	134.57	

Bold text indicates statistically significant results (p < 0.05).

** denote statistical significance: *p < 0.05, **p < 0.01.

NA, Not Applicable.

Lowercase letters refer to group differences determined by post hoc analysis.

### C-reactive protein average value trend

**Figure 3:**
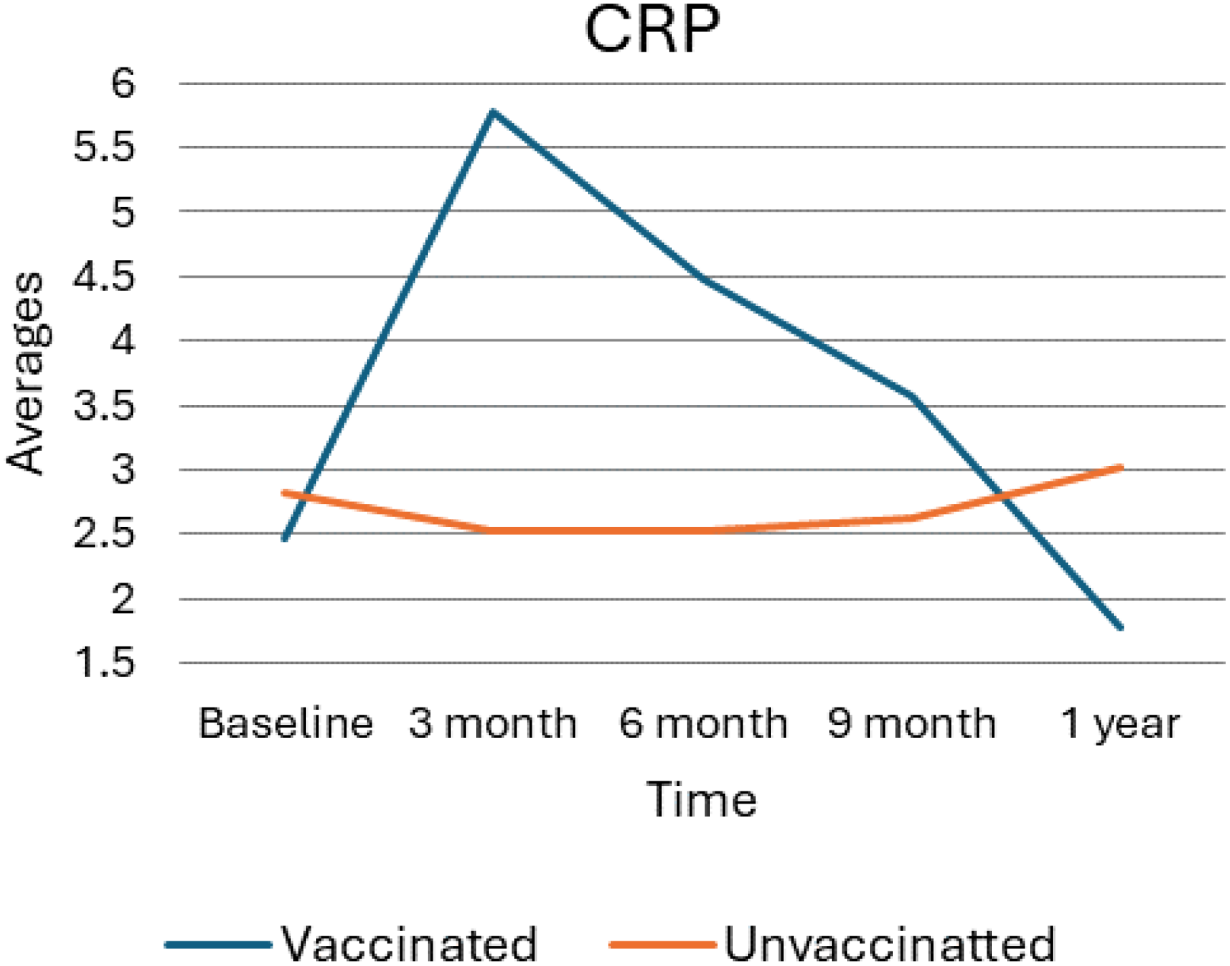
Average value for CRP levels for vaccinated and unvaccinated groups during various time points. The above Image illustrates the average CRP levels for both vaccinated and unvaccinated groups across various time points. The x-axis represents the follow-up time (baseline, 3 months, 6 months, and 9 months), whereas the y-axis displays the average CRP concentration. In general, the graph highlights the trend of CRP changes, showing an increase at 3 months followed by a decline.

As shown in the following **Figure 3**, the trend in C-reactive protein (CRP) levels among vaccinated and unvaccinated individuals over one year, with measurements taken at baseline, 3 months, 6 months, 9 months, and 1 year. After vaccination, CRP levels in the vaccinated group exhibit a sharp increase from baseline to 3 months, peaking at approximately 5.5 units. Following this rise, CRP levels show a steady decline, decreasing gradually over time and reaching approximately 2.5 units at the 1-year mark. Considerably, this 1-year level is slightly below the baseline measurement, indicating a return toward baseline levels. In contrast, the unvaccinated group displayed relatively stable CRP levels throughout the study period, with only minor fluctuations observed. At the 1-year mark, there is a slight increase in CRP levels, which rise slightly above 3 units, but overall, the levels remain constant compared to the baseline. The overall data suggest that vaccination leads to a temporary spike in CRP levels, indicative of an acute inflammatory response shortly after vaccination. This is followed by a gradual return to baseline levels as the immune response stabilizes over time. In contrast, CRP levels in unvaccinated individuals remain more consistent throughout the observed period, indicating a lack of similar inflammatory responses.

### GEE multiple regression of C-reactive protein – crude and adjusted effects

As **Table 6** shows, according to this model, both crude and adjusted models, vaccination status was a significant predictor of CRP levels, with vaccinated individuals showing lower CRP levels (Crude β = 0.90, 95% CI: 0.78, 1.02; p < 0.001; Adjusted β = 0.95, 95% CI: 0.84, 1.07; p < 0.001) compared to the unvaccinated group. Time also significantly influenced CRP levels, with reductions noted at 3 months. Analysis of demographic factors found that participants aged 40 years or younger had significantly lower CRP levels compared to those older than 40 years, in both crude (β = -0.85, 95% CI: -1.03, -0.66; p < 0.001) and adjusted models (β = -0.91, 95% CI: -1.03, -0.79; p < 0.001). Additionally, Female participants exhibited lower CRP levels compared to males in both the crude (β = -0.26, 95% CI: -0.45, -0.07; p = 0.008) and adjusted analyses (β = -0.29, 95% CI: -0.41, -0.18; p < 0.001). Furthermore, the effects of alcohol, chat, and smoking exposures on CRP levels were not statistically significant, indicating that these exposures did not independently affect CRP in the population studied. Overall, these findings suggest that vaccination status and time significantly influence CRP levels, with younger age and female sex also contributing to lower levels, while medication use and lifestyle factors had less impact.

**Table 6:** GEE multiple regression of C-Reactive Protein (CRP) – crude and adjusted effects.

Variables (N=224)	Crude Effect	Adjusted Effect
	β (95% CI)	P-Value
Vaccination Status		
Vaccinated	.90 (.78, 1.02)	<.001**
	.95 (.84, 1.07)	<.001*
Unvaccinated	Ref.	Ref.
**Time**		<.001**
3 months	.62 (.41,.83)	<.001**
6 months	.29 (.15,.43)	<.001*
9 months	.13 (.04,.22)	.004*
1 year	-.03 (-.09,.04)	.461
Baseline	Ref.	Ref.
Group*Time	Not Applicable	Not Applicable
Vaccinated * 3 months	Not Applicable	Not Applicable
Vaccinated * 6 months	Not Applicable	Not Applicable
Vaccinated * 9 months	Not Applicable	Not Applicable
Vaccinated * 1 year	Not Applicable	Not Applicable
Vaccinated * Baseline	Ref.	Ref.
Age Group		
<=40 years	-.85 (-1.03, -.66)	<.001**
	-.91 (-1.03, -.79)	<.001*
>40 years	Ref.	Ref.
Sex		
Female	-.26 (-.45, -.07)	.008**
	-.29 (-.41, -.18)	<.001*
Male	Ref.	Ref.
Alcohol Exposure		
Yes	.01 (-.24,.26)	.964
No	Ref.	Ref.
Chat Exposure		
Yes	.13 (-.19,.45)	.429
No	Ref.	Ref.
Smoking Exposure		
Yes	.22 (-.34,.79)	.442
No	Ref.	Ref.
**Medications**		.048**
Insulin	-.01 (-.29,.27)	.950
	.03 (-.13,.19)	.702
Metformin	.24 (.01,.46)	.044
	.08 (-.06,.22)	.273
Both	Ref.	Ref.
**Intercept**	2.97	

Bold text indicates statistically significant results (p < 0.05).

** denote statistical significance: *p < 0.05, **p < 0.01.

### interferon gamma average trend

**Figure 4:**
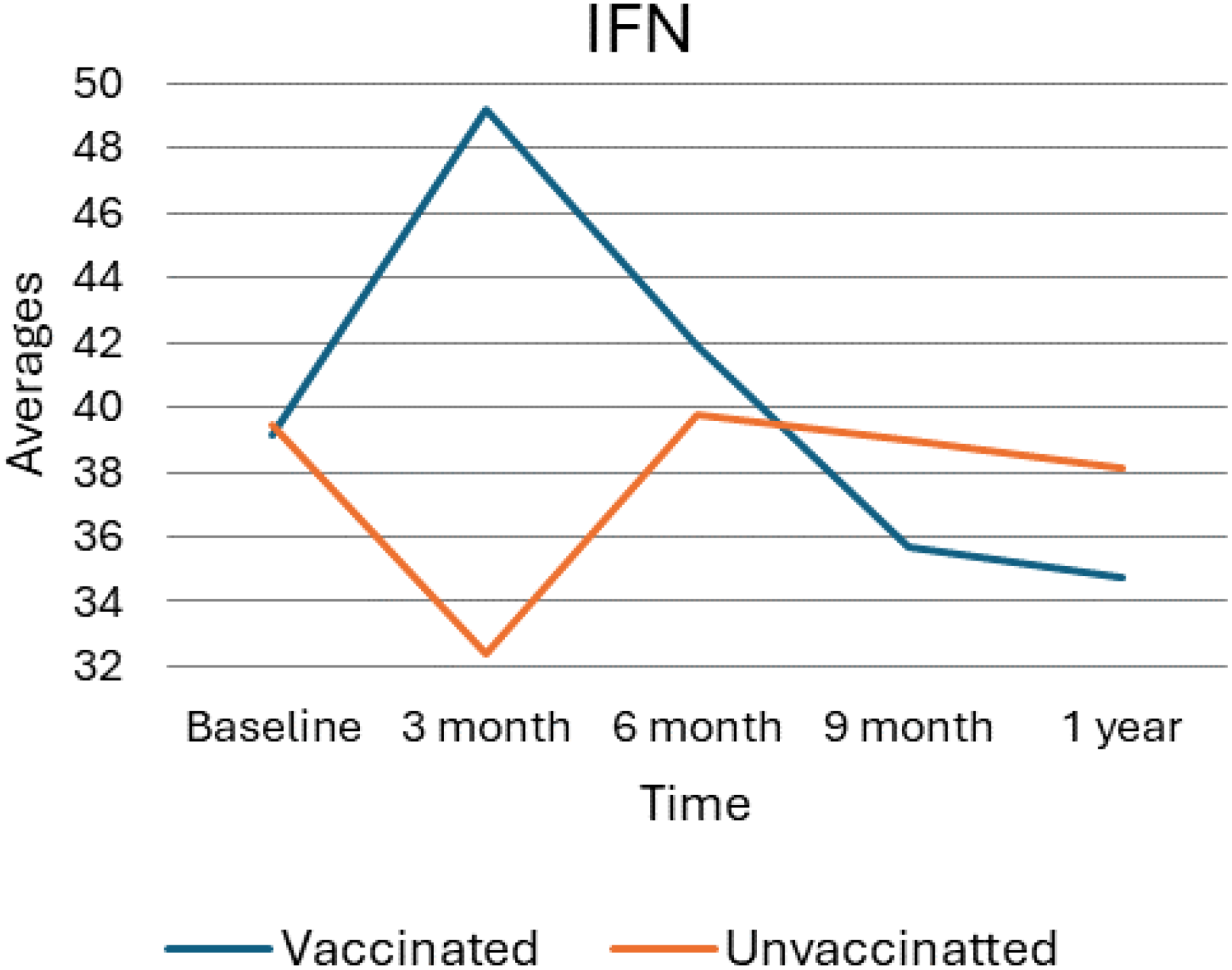
Average trend of interferon gamma values for unvaccinated and vaccinated groups over time. The above Image illustrates the average IFN-γ levels for both vaccinated and unvaccinated groups across various time points. The x-axis represents the follow-up time (baseline, 3 months, 6 months, and 9 months), whereas the y-axis displays the average IFN-γ concentration. In general, the graph highlights the trend of IFN-γ changes, showing an increase at 3 months followed by a decline).

As depicted in the following figure, (**Figure 4**) the average levels of Interferon (IFN) over time for vaccinated and unvaccinated groups were checked. Accordingly, at baseline, both groups begin at similar levels, and by the 3-month mark, there is a notable divergence: the vaccinated group exhibits a sharp increase in IFN levels, while the unvaccinated group shows a significant decrease. At six months, the vaccinated group’s IFN levels declined markedly, approaching levels like the unvaccinated group, which shows a slight increase during the same period. By nine months, the vaccinated group’s IFN levels continue to decrease, while the unvaccinated group stabilizes slightly. At the 1-year mark, both groups exhibit a downward trend, with the vaccinated group maintaining lower IFN levels compared to the unvaccinated group. Overall, this trend suggests a significant initial boost in IFN among the vaccinated group, followed by a decline over time, whereas the unvaccinated group shows more stable but consistently lower IFN levels.

### Generalized estimating equations multiple regression analysis for interferon-gamma

As presented in the following **Table 7**, the results of the Generalized Estimating Equations (GEE) multiple regression analysis for interferon-gamma (IFN-γ) levels among 224 participants, showcasing both crude and adjusted effects. The impact of vaccination status, time points, their interaction, age, sex, and various exposures (alcohol, chat, smoking), along with medication use on IFN-γ levels were analyzed and the crude analysis, vaccination status showed a significant effect with a β coefficient of 2.39 (95% CI: 0.00, 4.79; p = 0.050). Moreover, time was a significant predictor of IFN-γ levels in both the crude and adjusted models and significant changes were observed across various time points, with decreases at 3 months (adjusted β = -7.09, 95% CI: -8.63, -5.54; p < 0.001), increases at 6 months (adjusted β = 0.35, 95% CI: 0.20, 0.50; p < 0.001), and decreases at 9 months (adjusted β = -0.43, 95% CI: -0.61, -0.26; p < 0.001) and 1 year (adjusted β = -1.33, 95% CI: -1.51, -1.16; p < 0.001) compared to baseline, demonstrating significant temporal fluctuations in IFN-γ levels.

**Table 7:** GEE multiple regression of interferon-gamma (IFN-γ)– crude and adjusted effects.

Variables (N=224)	Crude Effect	Adjusted Effect
	β (95% CI)	P-Value
Vaccination Status		
Vaccinated	2.39 (.00, 4.79)	.050**
	-.26 (-2.62, 2.11)	.831
Unvaccinated	Ref.	Ref.
**Time**		<.001**
3 months	-2.73 (-4.26, -1.21)	<.001
	-7.09 (-8.63, -5.54)	<.001*
6 months	.96 (.77, 1.15)	<.001
	.35 (.20,.50)	<.001*
9 months	-1.22 (-1.44, -.99)	<.001
	-.43 (-.61, -.26)	<.001*
1 year	-2.12 (-2.34, -1.89)	<.001
	-1.33 (-1.51, -1.16)	<.001*
Baseline	Ref.	Ref.
Group*Time	Not Applicable	<.001*
Vaccinated * 3 months	17.09 (15.42, 18.77)	<.001*
Vaccinated * 6 months	2.39 (2.09, 2.71)	<.001*
Vaccinated * 9 months	-3.09 (-3.32, -2.86)	<.001*
Vaccinated * 1 year	-3.09 (-3.32, -2.86)	<.001*
Vaccinated * Baseline	Ref.	Ref.
Age Group		
<=40 years	-.15 (-2.16, 1.87)	.887
>40 years	Ref.	Ref.
Sex		
Female	1.91 (.02, 3.80)	.047**
	1.92 (.06, 3.79)	.044*
Male	Ref.	Ref.
Alcohol Exposure		
Yes	-.58 (-3.16, 1.99)	.657
No	Ref.	Ref.
Chat Exposure		
Yes	-.76 (-3.96, 2.44)	.641
No	Ref.	Ref.
Smoking Exposure		
Yes	3.01 (-2.04, 8.06)	.242
No	Ref.	Ref.
**Medications**		.750
Insulin	.98 (-1.56, 3.51)	.451
Metformin	.51 (-1.70, 2.73)	.650
Both	Ref.	Ref.
**Intercept**	38.45	

** denote statistical significance: *p < 0.05, **p < 0.01.

Bold text indicates statistically significant results (p < 0.05).

Additionally, age group analysis showed no significant effect, with individuals aged 40 years or younger not differing significantly from those older than 40 years (crude β = -0.15, 95% CI: -2.16, 1.87; p = 0.887; adjusted β = -0.15, 95% CI: -2.16, 1.87; p = 0.887). Sex had a significant effect in both models, with females exhibiting higher IFN-γ levels compared to males (crude β = 1.91, 95% CI: 0.02, 3.80; p = 0.047; adjusted β = 1.92, 95% CI: 0.06, 3.79; p = 0.044). Alcohol, chat, or smoking exposures and medication use did not significantly impact IFN-γ levels in the adjusted model.

### Interleukin 6 average value trend and effect size

**Figure 5:**
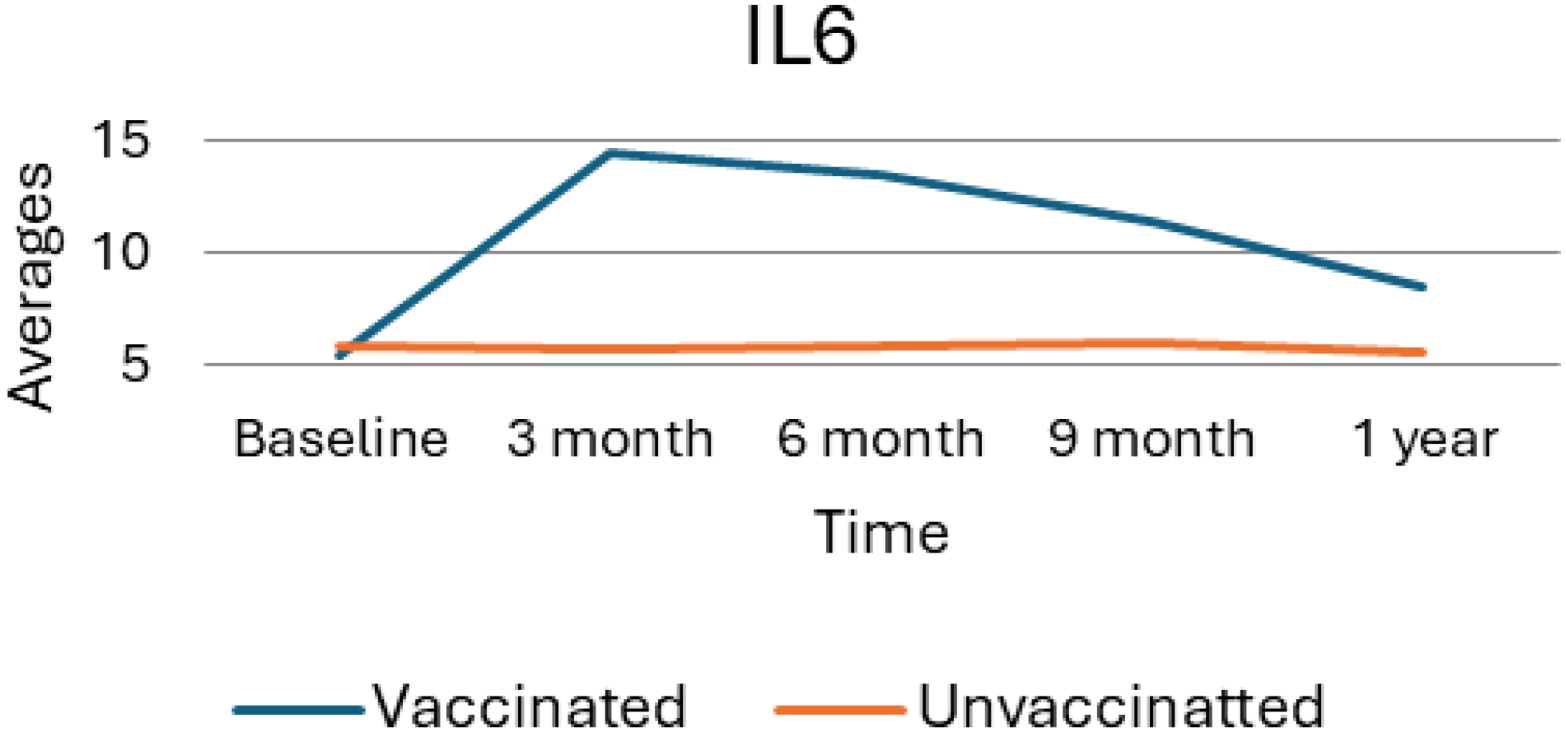
Average trend of interleukin 6 values for unvaccinated and vaccinated groups over time. The above Image illustrates the average IL6 levels for both vaccinated and unvaccinated groups across various time points. The x-axis represents the follow-up time (baseline, 3 months, 6 months, and 9 months), whereas the y-axis displays the average IL6 concentration. In general, the graph highlights the trend of IL6 changes, showing an increase at 3 months followed by a decline.

As **Figure 5** provides a visual representation of the average levels of IL6 over time, comparing the trends between vaccinated and unvaccinated groups. At baseline measurement, both vaccinated and unvaccinated groups exhibit similar IL6 levels indicating no significant differences at the outset of the study. However, vaccinated individuals show a slight upward trend in IL-6 levels from the baseline. As the result showed, the vaccinated group experienced a pronounced increase in IL-6 levels, peaking at the 3-month mark and reflecting a significant inflammatory response after vaccination, suggesting that the immune system is actively responding to the vaccine. Following the rise IL6 levels in the vaccinated group began to gradually decline indicating a decrease from the peak, suggesting a lingering immune response.

### Generalized estimating equations multiple regression analysis for interleukin 6

As shown in **Table 8**, generalized Estimating Equations (GEE) analysis provides significant insights into the factors influencing IL-6 levels among 224 participants based on their vaccination status, time since vaccination, and demographic factors. Accordingly, both crude and adjusted analyses reveal that vaccinated individuals exhibit significantly higher IL-6 levels compared to their unvaccinated counterparts. The crude β value is 4.89 (95% CI: 4.54, 5.25) and the adjusted β value is 4.96 (95% CI: 4.64, 5.29), with p-values <.001 for both analyses and strong association underscores the immunological response elicited by vaccination.

**Table 8:** GEE multiple regression of interleukin 6 (IL-6) _crude and adjusted effects.

Variables (N=224)	Crude Effect	Adjusted Effect
	β (95% CI)	P-Value
Vaccination Status		
Vaccinated	4.89 (4.54, 5.25)	<.001**
	4.96 (4.64, 5.29)	<.001*
Unvaccinated	Ref.	Ref.
**Time**		<.001**
3 months	2.17 (1.62, 2.71)	<.001
	2.17 (1.62, 2.71)	<.001*
6 months	1.98 (1.49, 2.46)	<.001
	1.98 (1.49, 2.46)	<.001*
9 months	1.62 (1.26, 1.98)	<.001
	1.62 (1.26, 1.98)	<.001*
1 year	.56 (.33,.78)	<.001
	.56 (.33,.78)	<.001*
Baseline	Ref.	Ref.
Group*Time	Not Applicable	Not Applicable
Vaccinated * 3 months		
Vaccinated * 6 months		
Vaccinated * 9 months		
Vaccinated * 1 year		
Vaccinated * Baseline	Ref.	Ref.
Age Group		
<=40 years	-1.07 (-1.73, -.42)	.001**
	-1.30 (-1.52, -1.07)	<.001*
>40 years	Ref.	Ref.
Sex		
Female	-.18 (-.79,.44)	.573
Male	Ref.	Ref.
Alcohol Exposure		
Yes	.25 (-.54, 1.04)	.537
No	Ref.	Ref.
Chat Exposure		
Yes	.62 (-.51, 1.75)	.282
No	Ref.	Ref.
Smoking Exposure		
Yes	.95 (-.95, 2.85)	.326
No	Ref.	Ref.
**Medications**		.638
Insulin	-.22 (-1.24,.79)	.667
Metformin	-.37 (-1.15,.41)	.354
Both	Ref.	Ref.
**Intercept**	4.91	

Bold text indicates statistically significant results (p < 0.05).

** denote statistical significance: *p < 0.05, **p < 0.01.

Moreover, age plays a significant role in IL-6 responses, and individuals aged 40 years or younger exhibit lower IL-6 levels compared to those over 40. Additionally, factors such as sex, alcohol, chat, and smoking exposure, as well as medication types (insulin and metformin), do not show a significant impact on IL-6 levels. Overall, these results highlight the significance of vaccination status, time since vaccination, and age in influencing IL-6 levels, while other demographic and lifestyle factors appear to be negligible.

## Discussion

This study has indicated that IgG levels did not differ significantly long-term between vaccinated and unvaccinated groups. The vaccine might appear to cause some initial immune response, yet in diabetic patients, this was not sustained for the long term. This is a particular concern regarding long-term immunity and protection against reinfection since IgG normally plays such an important role. In the absence of its sustained elevation, the durability of the immune response may be missed in this population. These findings contrast with the previous studies, especially those with mRNA vaccines, in which IgG levels were kept up even long after vaccination. This may be because diabetic patients have specific physiological challenges and cannot sustain this production over time due to impairment in basic metabolisms and immune systems (8).

Studies showed that diabetes can have a significant impact on our immune system, affecting how our bodies respond to vaccines. For people with diabetes, the immune system often doesn’t work as well due to complications affecting innate immune cells like macrophages and dendritic cells. This can make it harder for the body to recognize pathogens and present antigens, which are essential steps for kickstarting the adaptive immune response (9). Additionally, recent studies indicated that high blood sugar levels and insulin resistance can weaken T-cells, which play a critical role in our body’s defense against infections, leading to a less effective cellular immune response. It was also found that people with diabetes also struggle with humoral immunity, experiencing lower antibody production after vaccination because of issues with B-cell function (10).

Other studies also suggest that the booster dose, when administered, might become particularly significant for diabetic patients to retain sufficient IgG levels (11) and offer long-term protection against COVID-19 and other similar infections (12). In this respect, Munoz and his colleagues recommended the urgent need for adaptive vaccination strategies in this population of vulnerable individuals with diabetes mellitus. Overall, these results confirm the necessity for follow-up monitoring of IgG levels in patients with diabetes post-vaccination. Moreover, this research can also hint at the need for further doses or boosters for maintaining immunity appropriately in these patients. By understanding the weaknesses of the initial immune response, healthcare policy makers can strategize with more insight regarding vaccination protocols and management to enhance protection against reinfection in diabetic patients (13).

The much-heightened IgM at three months post-vaccination (p = 0.004) identified in diabetic patients who were vaccinated with the Ad26.COV2.S indicated a very strong early immune response. IgM is the first antibody produced upon exposure to a pathogen; as such, one would expect early elevation of IgM, indicative of a very effective primary immune response that the vaccine elicits (14). IgM decreases over time and starts well in line with the unvaccinated group at nine months, reflecting a transient humoral response. This may indicate a possible perturbation in the long-term immunity amongst this population. IgM response followed a pattern that showed that vaccinated individuals were raised shortly after vaccination and then gradually subsided (15). This is usually what happens when IgG starts its production and takes over the function of IgM during the infection period. This may reflect that the initial rise, followed by a rapid decline of IgM, in diabetic patients who are usually immunocompromised, has the ability of the immune system to sustain a long response somewhat compromised, and therefore, booster doses may be required for adequate and sustained protection (16).

Comparing this to other studies, which show that the peak in IgM does not necessarily relate to diminished protection, provided IgG levels remain high enough; a lack of sustained IgM levels in the present study may reveal potential vulnerability to reinfection (17). This is of special concern within the diabetic population, which often already acts as a host to an impaired immune response (4).

The dramatic increase in CRP levels from three months post-vaccination, at a p-value of less than 0.001, indicates the aftermath of the acute inflammatory response of the administration of the Ad26.COV2.S (18). Highly increased levels of CRP in general indicate inflammation in the body and are therefore considered a marker of the body’s immune response to the vaccine antigen. After this peak, the level further declined gradually, even lower than pre-vaccination levels, one-year post-vaccination (19).

This elevation in CRP following vaccination agrees with observations made in other research, especially among subjects with predispositions such as diabetes. Other studies also reported increased CRP levels following COVID-19 vaccination among diabetic patients, which they attributed to the pro-inflammatory action of the vaccination. This may be more pronounced in cases with a predisposition to inflammatory disorders (20).

This study has also shown that CRP levels decrease accordingly after this initial elevation, and this is considered encouraging because this means the inflammatory response does not advance to chronic inflammation-which is very risky for developing other complications in diabetic patients too (21). The CRP response following the Ad26.COV2.S (Janssen COVID-19 Vaccine)vaccine administration is consistent compared with other vaccines, though probably a little stronger due to its adenoviral vector platform. It is known to lead to higher inflammatory responses compared to mRNA vaccines. The pattern observed in CRP levels in this study indeed indicates that the vaccine activates the immune system (22).

This resolution of inflammation is very important in diabetic patients, as chronic states of inflammation result in poor health outcomes as per Williams et al. (23). The study showed a significantly higher IFN-γ level at three months post-vaccination (p < 0.001), which then gradually reduced. IFN-γ is a critical cytokine that has been essentially involved in immune defense, especially antiviral responses (3). The temporal peak of IFN-γ presented after vaccination signifies intense cellular-mediated immunity soon after vaccination, crucial for maintaining viral replication below levels that can give long-term immunity. These results agree with other studies that similarly showed high levels of IFN-γ after vaccination with adenoviral vectors. This indicates that the adenoviral vectors, such as the Ad26.COV2.S (Janssen COVID-19 Vaccine) caused a Th1-biased immune response since it would be largely ideal in combating an infection caused by a virus (20). However, the trend of observed IFN-γ levels through time has indeed rung an alarm at one year with a significant decline in this cytokine level, thus raising an important question about the cellular immune response durability in diabetic patients. This fall in IFN-γ levels may indicate that subjects in this population could need additional booster doses and specific approaches for diabetic patients to maintain good protective immunity throughout (18).

Comparative studies have elucidated that while IFN-γ levels normally decrease after the acute phase of immune activation, maintaining it at a higher level is even more crucial for immunocompromised patients, as evidenced by diabetic patients. It has been observed that, while the Ad26.COV2.S (Janssen COVID-19 Vaccine)might indeed provoke an early IFN-γ response, the fact that there may be a need for booster doses underscores one of the most important aspects of ongoing vaccine strategies as related to this vulnerable population (24).

This study showed that the levels of IL-6 increased significantly by three months after vaccination, peaking at this time before a gradual decline. IL-6 is a crucial cytokine in inflammation and the acute phase response; hence, the pattern seen here shows that the Ad26.COV2.S (Janssen COVID-19 Vaccine)indeed provokes a strong initial inflammatory response necessary for immune activation. This finding is in good agreement with reports of post-vaccination elevations of IL-6, particularly in individuals suffering from chronic inflammatory diseases such as diabetes (25).

A study indicated that the elevation of IL-6 is temporary and already started to trend toward baseline level values at one one-year time point, reflecting a well-regulated inflammatory response to vaccination. Such regulation decreases the risk of chronic inflammation, which is highly common and may contribute to further complications in diabetic patients (24).

Chronic inflammation and metabolic issues are common in people with diabetes and can really affect how their immune system works, especially after getting vaccinated. Diabetes tends to be linked with ongoing low-grade inflammation (26). This means that there are higher levels of certain substances in the body, like IL-6 and CRP, that signal inflammation. This persistent state of inflammation can hinder the performance of immune cells, such as T-cells and macrophages, which are crucial for fighting off infections and responding to vaccines (27).

Additionally, issues like insulin resistance and high blood sugar, which often accompany diabetes, can interfere with how immune cells communicate and present antigens (28). This disruption might contribute to the weaker and sometimes shorter immune responses seen in diabetic patients, especially with vaccines like the Ad26.COV2.S (Janssen COVID-19 Vaccine). Essentially, if the immune system isn’t functioning at its best, the effectiveness of the vaccine could be impacted. Recognizing these connections is important for creating vaccination strategies tailored specifically for people with diabetes to help improve their immune response and overall health (29).

These disturbances in the immune response are a result of a mix of metabolic and inflammatory factors, which may explain why those with diabetes often have a less robust immune protection after getting vaccinated. Understanding these challenges is important for developing better vaccination strategies tailored to the needs of individuals with diabetes. effective, tailored vaccination strategies for diabetic populations (30).

In general, the observed trend in this study suggests that the Ad26.COV2.S (Janssen COVID-19 Vaccine)effectively activates the immune system while ensuring that the inflammatory response does not become excessive or prolonged. This balance is crucial for the safety and well-being of diabetic patients, who may be more susceptible to the adverse effects of prolonged inflammation.

## Implications and lessons learned

Our study indicated the need for critical considerations for vaccination of diabetic patients, a population with unique and special immunological and physiological vulnerabilities. Diabetes is associated with altered immune responses, chronic inflammation, and increased susceptibility to various infections, making it imperative to understand how vaccines interact with their immune systems. The immunological response to the Ad26.COV2.S (Janssen COVID-19 Vaccine)observed in this cohort underscores the need for tailored vaccine strategies for diabetic populations, particularly during pandemics and outbreaks of emerging infectious diseases.

Our study findings of immunological response in diabetic patients included larger cohorts of individuals with chronic conditions. This will ensure that vaccines are tailored and optimized for these populations and provide robust protection. Additionally, this study highlights the need to consider booster strategies to address potentially weaker immune responses in diabetic individuals. Although COVID-19 is transitioning into an endemic phase, the insights gained from this study are relevant for other viral infections and vaccines targeting immunocompromised populations. These findings serve as a blueprint for addressing similar challenges in vaccine research and delivery, particularly for diseases where chronic conditions exacerbate risks.

## Conclusions

In conclusion, the vaccine elicited a significant but transient immunological response in diabetic patients. The study suggests the need for booster doses and ongoing monitoring of immune responses in this population. However, the observed decline in sustained IgG levels indicates that the long-term immunological benefits may be limited in this vulnerable population. These findings emphasize the importance of considering booster doses to enhance protective immunity and suggest the need for ongoing monitoring of immune responses in diabetic patients. Such strategies are critical to ensuring continued protection against vaccine-preventable diseases and addressing the specific health needs of individuals with underlying conditions. By adopting a proactive approach to vaccination and immune response management, we can better safeguard the health of this at-risk group. We emphasize the need for further studies on booster doses for diabetic patients. Looking ahead, there’s a lot of potential for research to focus on vaccine formulations specifically designed for people with diabetes. It would be great to explore a variety of vaccine types, such as mRNA and viral vector vaccines, to see which might work best. Another important area could be adjusting the timing of vaccine doses to better match how individuals with diabetes respond, ensuring that everyone gets the most effective protection possible.

## Study limitations and future directions

Although this study incorporates statistical adjustments to address differences between the vaccinated and unvaccinated groups, the possibility of selection bias cannot be eliminated. Future research would benefit from the implementation of propensity score matching (PSM) to improve the comparability of the groups by effectively balancing observed covariates. Furthermore, we recognize that conducting additional sensitivity analyses could yield more comprehensive insights into the robustness of our findings. While our present analytical strategy is statistically valid, employing these advanced methodologies could significantly enhance the rigor and precision of subsequent investigations in this field.

## Data Availability

The raw data supporting the conclusions of this article will be made available by the authors, without undue reservation.

## References

[1] Lima-Martínez MM Carrera Boada C Madera-Silva MD Marín W Contreras M. COVID-19 and diabetes: A bidirectional relationship. Clin Invest Arterioscler. (2021) 33:151–7. doi: 10.1016/j.artere.2021.04.004 PMC7598432 33303218

[2] Khunti K Valabhji J Misra S. Diabetes and the COVID-19 pandemic. Diabetologia. (2023) 66:255–66. doi: 10.1007/s00125-022-05833-z PMC9685151 36418578

[3] Sahin U Muik A Derhovanessian E Vogler I Dormitzer PR Jansen KU. COVID-19 vaccine BNT162b1 elicits human antibody and TH1 T cell responses. Nature. (2020) 586:594–9. doi: 10.1038/s41586-020-2814-7 32998157

[4] Chen F Zhong Y Li J Luo J. Dynamic changes of SARS-CoV-2 specific IgM and IgG among population vaccinated with COVID-19 vaccine. Epidemiol Infect. (2022) 150:1–17. doi: 10.1017/S0950268822001388 PMC9050050 35392994

[5] Rikitu Terefa D Shama AT Feyisa BR Ewunetu Desisa A Geta ET Chego Cheme M. COVID-19 vaccine uptake and associated factors among health professionals in Ethiopia. Infect Drug Resist. (2021) 19:5531–41. doi: 10.2147/IDR.S344647 PMC8702775 34984008

[6] Zhu H Liu S Zheng W Belay H Zhang W Qian Y. Assessing the dynamic impacts of non-pharmaceutical and pharmaceutical intervention measures on the containment results against COVID-19 in Ethiopia. PloS One. (2022) 17:5050–9. doi: 10.1371/journal.pone.0271231 PMC9321453 35881650

[7] Diggle PJ Liang KY Zeger SL. Analysis of Longitudinal Data. 2nd ed. New York: Oxford University Press (1994). doi: 10.1198/tech.2003.s147

[8] Landau LM Kagan JC. ARIES domains: functional signaling units of type I interferon responses. FEBS J. (2025) doi:10.1111/febs.70023 PMC12353150 39964808

[9] Daryabor G Atashzar MR Kabelitz D Meri S Kalantar K. The effects of type 2 diabetes mellitus on organ metabolism and the immune system. Front Immunol. (2020) 11:158. doi: 10.3389/fimmu.2020.0158 32793223 PMC7387426

[10] Vaibhav Nishad SS Dongare D Tripathi ACP Tripathi T Tripathi P. Deciphering the intricacies of immune system dysfunction and its impact on diabetes mellitus: Revisiting the communication strategies to manage diabetes mellitus. Health Sci Rev. (2024) 13:100201. doi: 10.1016/j.hsr.2024.100201

[11] Anderson EJ Creech CB Berthaud V Piramzadian A Johnson KA Zervos M. Evaluation of mRNA-1273 Vaccine in Children 6 Months to 5 Years of Age. N Engl J Med. (2022) 387:1673–87. doi: 10.1056/NEJMoa2209367 PMC9634866 36260859

[12] van den Berg JM Remmelzwaal S Blom MT van Hoek BACE Swart KMA Overbeek JA. Effectiveness of COVID-19 Vaccines in Adults with Diabetes Mellitus: A Systematic Review. Vaccines. (2023) 11, 24. doi: 10.3390/vaccines11010024 PMC9861646 36679869

[13] Muñoz FM Cramer JP Dekker CL Dudley MZ Graham BS Gurwith M. Vaccine-associated enhanced disease: Case definition and guidelines for data collection, analysis, and presentation of immunization safety data. Vaccine. (2021) 39:3053–66. doi: 10.1016/j.vaccine.2021.01.055 PMC7901381 33637387

[14] Mantovani A Rescigno M Forni G Tognon F Putoto G Ictho J. COVID-19 vaccines and a perspective on Africa. Trends Immunol. (2023) 44:172–87. doi: 10.1016/j.it.2023.01.005 PMC9832054 36709083

[15] Dalla Gasperina D Veronesi G Castelletti CM Varchetta S Ottolini S Mele D. Humoral and cellular immune response elicited by the BNT162b2 COVID-19 vaccine booster in elderly. Int J Mol Sci. (2023) 24:193–204. doi: 10.3390/ijms241813728 PMC10530943 37762029

[16] Azkur AK Akdis M Azkur D Sokolowska M van de Veen W Brüggen MC. Immune response to SARS-CoV-2 and mechanisms of immunopathological changes in COVID-19. Allergy. (2020) 75:1564–81. doi: 10.1111/all.14364 PMC7272948 32396996

[17] Almehamadi Allahyani M Eed E Alharbi AM Halawi M Allam HH. Seroprevalence of igM and igG against SARS-coV-2 after two doses of pfizer-bioNTech COVID-19 vaccine in women with breast cancer. Clin Lab. (2022) 68:1403–8. doi: 10.7754/clin.lab.2022.220316 36378000

[18] Karimi A Shobeiri P Kulasinghe A Rezaei N. Novel Systemic Inflammation Markers to Predict COVID-19 Prognosis. Front Immunol. (2021) 12:741061. doi: 10.3389/fimmu.2021.741061 PMC8569430 34745112

[19] Ostrowski SR Søgaard OS Tolstrup M Stærke NB Lundgren J Østergaard L. Inflammation and platelet activation after COVID-19 vaccines- possible mechanisms behind vaccine-induced immune thrombocytopenia and thrombosis. Front Immunol. (2021) 54. doi: 10.3389/fimmu.2021.779453 PMC8649717 34887867

[20] Follegatti PM Gilbert SC Pollard AJ Oxford COVID Vaccine Trial Group. Safety and immunogenicity of the ChAdOx1 nCoV-19 vaccine against SARS-CoV-2: a preliminary report of a phase 1/2, single-blind, randomised controlled trial. Lancet. (2020) 396:467–78. doi: 10.1016/S0140-6736(20)31604-4 PMC7445431 32702298

[21] Tormo N Navalpotro D Serano M Moreno M Grosson F Gimeno C. Commercial Interferon-gamma release assay to assess the immune response to first and second doses of mRNA vaccine in previously COVID-19 infected versus uninfected individuals. Diagn Microbiol Infect Dis. (2022) 102:115573. doi: 10.1016/j.diagmicrobio.2021.115573 35121268 PMC8502494

[22] Langgartner D Winkler R Brunner-Weisser J Rohleder N Jarczok MN Gündel H. COVID-19 vaccination exacerbates ex vivo IL-6 release from isolated PBMCs. Sci Rep. (2023) 13(1):9496. doi: 10.1038/s41598-023-35731-2 PMC10261110 37308487

[23] Williams NL Nguyen THH Del Chiappa G Fedeli G Wassler P. COVID-19 vaccine confidence and tourism at the early stage of a voluntary mass vaccination campaign: A PMT segmentation analysis. Curr. Issues Tour. (2021) 25:475–89. doi: 10.1080/13683500.2021.1963216

[24] Schwake C Pakeerathan T Kleiter I Ringelstein M Aktas O Korporal-Kuhnke M. Humoral COVID-19 vaccine response in patients with NMOSD/MOGAD during anti-IL-6 receptor therapy compared to other immunotherapies. Mult Scler. (2023) 29:757–61. doi: 10.1177/13524585221151124 PMC9908518 36748649

[25] Cheong JG Ravishankar A Sharma S Josefowicz SZ. Epigenetic memory of coronavirus infection in innate immune cells and their progenitors. Cell. (2023) 186:3882–902. doi: 10.1016/j.cell.2023.07.019 PMC10638861 37597510

[26] Ayzenberg I. Humoral COVID-19 vaccine response in patients with NMOSD/MOGAD during anti-IL-6 receptor therapy compared to other immunotherapies. Mult Scler. (2023) 29:757–61. doi: 10.1177/13524585221151124 PMC9908518 36748649

[27] Talamandris S Antonopoulos AS Oikonomou E Papamikrouis GA Vogiatzi G Papaioannou S. The role of inflammation in diabetes: current concepts and future perspectives. Eur Cardiol. (2019) 14:50–9. doi: 10.15420/ecr.2018.33.1 PMC6523054 31131037

[28] Nishiyama T Miyamatsu Y Park H Nakamura N Yokokawa Shibata R Iwami S. Modeling COVID-19 vaccine booster-elicited antibody response and impact of infection history. Vaccine. (2023) 41:7655–62. doi: 10.1016/j.vaccine.2023.11.040 38008663

[29] Berbudi A Rahmadika N Tjahjadi AI Ruslami R. Type 2 diabetes and its impact on the immune system. Curr Diabetes Rev. (2020) 16:442–9. doi: 10.2174/1573399815666191024085838 PMC7475801 31657690

[30] He YF Ouyang J Hu XD Wu N Jiang ZG Bian N. Correlation between COVID-19 vaccination and diabetes mellitus: A systematic review. World J Diabetes. (2023) 14:892–918. doi: 10.4239/wjd.v14.i6.892 37383586 PMC10294060

